# How Safe Do Teenagers Behave on Facebook? An Observational Study

**DOI:** 10.1371/journal.pone.0104036

**Published:** 2014-08-27

**Authors:** Ellen Vanderhoven, Tammy Schellens, Martin Valcke, Annelies Raes

**Affiliations:** Department of Educational Studies, Ghent University, Gent, Belgium; Institut Pluridisciplinaire Hubert Curien, France

## Abstract

The substantial use of social network sites by teenagers has raised concerns about privacy and security. Previous research about behavior on social network sites was mostly based on surveys and interviews. Observational research overcomes problems inherent to this research method, for example social desirability. However, existing observational research mostly focuses on public profiles of young adults. Therefore, the current observation-study includes 1050 public and non-public Facebook-profiles of teenagers (13–18) to investigate (1) what kind of information teenagers post on their profile, (2) to what extent they protect this information using privacy-settings and (3) how much risky information they have on their profile. It was found that young people mostly post pictures, interests and some basic personal information on their profile. Some of them manage their privacy-settings as such that this information is reserved for friends' eyes only, but a lot of information is accessible on the friends-of-friends' pages. Although general risk scores are rather low, more detailed analyses show that teenagers nevertheless post a significant amount of risky information. Moreover, older teenagers and girls post more (risky) information while there are no differences in applying privacy settings. We found no differences in the Facebook behavior of teenagers enrolled in different education forms. Implications of these results are discussed.

## Introduction

In the current cyber society, new participatory platforms for communication are rapidly evolving. Social network sites (SNSs) are an expression of these new communication technologies, also called online social networks. In about five years, Facebook evolved from a SNS reaching only one college community to the most popular SNS with millions of users all over the world [1]. This growth is exemplary for the increasing popularity of SNSs in general, with both young and older users. Research shows that in Europe 73% of the 13–14 year olds and 82% of the 15–16 year olds now have a profile on a SNS [2].

This increasing popularity raises some concerns about privacy and security, since SNSs are based on providing personal information to connect and communicate with others. Due to these raising concerns, research has been set up to study how young people behave on SNSs. However, the existing research is mainly based on surveys and interviews, which reflects many deficiencies, for example social desirability [3]. Research based on alternative designs – such as observational research – remains rather scarce and mainly focuses on public profiles of young adults. To counter these shortcomings, an observational study of public and non-public (i.e., visible by friends or friends-of-friends) Facebook-profiles of young adolescents (13–18) was conducted, trying to map the way they behave on SNSs and whether this entails risks. Moreover, we tried to identify possible individual differences between users of different age, gender and education form.

### Content of SNS-profiles

In a survey based study, it was found that American teenagers put a variety of personal things on their profile; the most common things are their first name (82%) and pictures of themselves (79%) [4]. Next, 29% post their last name, 66% include pictures of friends, 61% include their city or town and 29% include videos. Other researchers found comparable results with Belgian teenagers, except for the higher amount of posted videos (37%) and last names (46%) [5]. The latter is probably caused by the growing popularity of Facebook – currently the most popular SNS [1] - in which the use of a last name is mandatory. These researchers also focused on a typical functionality of Facebook, namely *liked links* (which can be collected by pressing the I like-button), which 17% of the questioned teenagers incorporated in their profile [5]. Posting comments on other users' walls and posting pictures has also been found to be very popular among teens [6].

Only limited research focuses on differences in the content of profiles considering age, education or gender. Regarding these demographic variables, it was found that older teenagers (15–17 years old) tend to post more pictures and other personal information on their profile [4]. Girls post more pictures, while boys give more contact information. These findings were confirmed in a survey research involving Flemish teenagers [7]. Additionally, it was found there are no differences related to users being enrolled in different education forms in sharing general descriptive information, but pupils enrolled in vocational education and technical education share more contact information than those enrolled in general education [7].

### Privacy settings

While young adults (18–19) put all kinds of content on their Facebook profile, most of them also reported to have changed their privacy settings to some extent [8]. However, other researchers found that still 31% of their respondents - college undergraduates - did never change their privacy settings [9]. Similar results were found in a survey study involving younger children (9–16), with 29% sustaining a public profile or not knowing about their privacy settings and 28% opting for partially private settings so that friends-of-friends could see their page [2]. While friends-of-friends suggests a friendship-based relationship, these people are nevertheless mostly strangers. This is especially the case considering that 46% of the children being questioned, accepted people as *friends* they met on the Internet and did not know face-to-face [2].

Furthermore, it was found that while older teens tend to make more personal information available [4], they are not more likely to adopt more stringent privacy settings [2]. Additionally, girls tend to change their privacy settings more than boys [2], [10].

### Risky behavior on SNSs

When talking about risky behavior on a SNS profile, most authors focus on the disclosure of personal information, allowing the viewer of the profile to identify and contact the profile owner, and on the use of privacy settings [2], [11], [12]. Indeed, a vast amount of studies find that teenagers post a lot of personal information on their profile and do not use privacy settings (see above). Unintended consequences of revealing these sorts of *risky information* include damaged reputation, gossip, stalking, identity-theft and the use of personal information by third parties like advertisers or by superiors like teachers [9], [13].

However, next to revealing personal information, revealing other types of information can be recognized as risky behavior as well, such as revealing information that could compromise teenagers' safety or that could lead to problematic outcomes [14]–[16]. Examples of these sorts of information are cyberbullying related messages, or pictures that demonstrate alcohol and drug abuse [17]. A survey study indicated that 20% of the adolescents with a SNS-profile published profile items they would not want current or prospective employers to see (mostly alcohol-related pictures or comments [18]). Moreover, 18% of publicly available MySpace profiles of adolescents showed evidence of alcohol use, 5% included pictures in swimsuit or underwear and 33% included swear words in their comments [19]. In a more recent survey, it was found that 17% of the participants posted pictures on their profile in which they can be seen drinking alcohol [20].

Furthermore, as already stated, the likelihood of providing personal information increases with age [4] and boys tend to disclose more personal information than girls [5]. Additionally, boys share significantly more self-promoting and risky pictures or comments (involving sex or alcohol), while girls were more likely to post romantic or cute pictures and information [18]. Moreover, pupils enrolled in vocational and technical education might be more vulnerable, as they share more contact information [7].

The behavior as revealed in the previous studies may reflect a threat, since [20] the exposure of personal information on SNSs is indeed associated with negative online experiences [21]. As they only focused on personal information in general, we can assume the consequences to be even worse when publicly exposing risky information related to alcohol and drug abuse, pictures in underwear, signs of aggression, etc. Indeed, exposure/unintentional disclosure of information or pictures is one of the four main reasons that adolescents report to have had bad experiences on Facebook [14].

### Surveys versus Observation

As stressed earlier, most available SNS research involving teenagers is based on self-report measures [21]. Due to the nature of these studies, available information about SNS profiles, the nature of privacy settings, and the level of risk behavior might be biased resulting in a low reliability and validity. Indeed, pupils might have given wrong answers, either because of social desirability [3] or because they do not know the right answer. Researchers emphasize that teenagers' mental model of their privacy settings does not always match the actual settings [8].

Above research drawbacks can be overcome by observing and analyzing teenagers' SNS profiles, so that the information can be coded objectively. Moreover, an observational approach gives the possibility to gather more detailed information about the amount and the nature of the content found online. However, due to practical reasons, this kind of studies is rather scarce in available literature. Moreover, the few studies available building on a content analysis of observed profiles, mainly focused on particular information types, for example profile pictures [16], [22] or on publicly available profiles (e.g., [12], [19], [23]). Since Facebook incorporates the safety precaution that minors can only share their profile with friends-of-friends, teenagers' profile pages on Facebook are non-public by design (but not private, as it can be visible for friends-of-friends, i.e. possible strangers). Therefore, observational research about the behavior of adolescents on SNSs has mainly focused on undergraduate students, a rather accessible research population in academic contexts [21]. As a result, it is difficult to come to decisive conclusions about currently applied privacy settings or the amount and nature of risky behavior of teenagers. Information of these younger users is however especially interesting considering the fact this behavior is shaped at an earlier age and in view of the development of appropriate education about SNSs.

For this reason, the current observational study extends the results found in previous observational research by focusing on Facebook-profiles of 13 to 18-year olds. The study aimed at answering the following research questions: (1) What kind of information do teenagers post on their Facebook-profile page? (2) Do teenagers manage privacy settings to secure this information? and (3) Does the available information entail particular risks? Additionally, for every research question individual differences based on age, gender and education form were explored.

## Method

### Procedure

**Figure 1:**
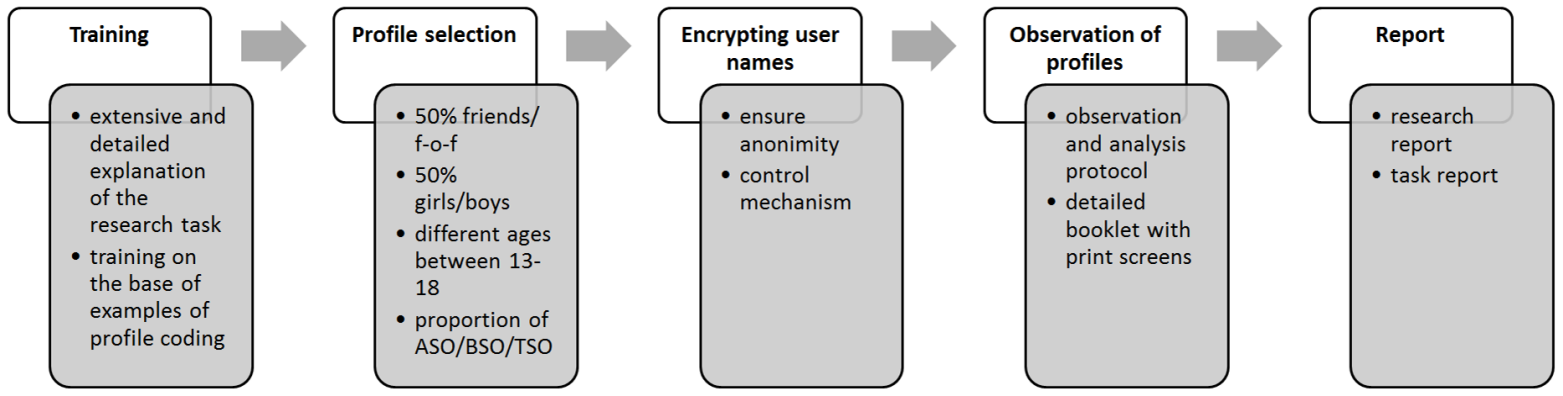
Sequential steps of the research procedure.

The research procedure is depicted in Figure 1 and explained below. Next to the main researchers, a large group of research-assistants were involved in the study. These received an extensive training on how to code profiles using a detailed codebook. The different steps of the analysis procedure were explained extensively. The stringent protocol could also be found on a website, continuously accessible after the training. Moreover, a codebook with print screens of coded example profiles and clear instructions were handed over to all trained research-assistants.

Secondly, profile pages were selected. Since we wanted to extend previous research by collecting information about non-public profiles on Facebook, we needed a more complex sampling method. Pages of friends and friends-of-friends can only be seen by friends and friends-of-friends, and not by the main researchers. Our sampling method overcame this problem by involving 179 research-assistants as observers in this study (more information about these research-assistants can be found in Appendix S1). These research-assistants were randomly divided into groups of four. Every group carried out the observational analysis of 24 Facebook profile pages of Flemish teenagers. All groups picked 12 profiles of their friends and 12 profiles of friends-of-friends, following a stratified random sampling procedure with age, gender and education form as strata.

The selection procedure resulted in the analysis of 1050 Facebook profiles. The final sample reflected a proportional participation of gender (49% boys, 51% girls), age levels (30% 13–14, 35% 15–16, and 35% 17–18 years old) and education forms as found in the Flemish secondary school population (Table 1).

**Table 1:** Comparison of our sample and the Flemish population with regard to education form.

	Sample	Flemish population*
General secondary education (ASO)	47%	41%
Technical secondary education (TSO)	31%	31%
Vocational secondary education (BSO)	19%	26%
Art education (KSO)	3%	2%

*[37].

After profile selection, user names were transformed using an encrypting tool made available on a specific research website. This guaranteed anonymity of profile owners and also prevented unintentional double inclusion of the same user profile.

After profile user encryption, the observational analysis took place on the base of a detailed codebook. All information on a user profile was coded by determining the type of information – pictures, videos, contact information -and the extent to which this information was available. Finally, all groups of researcher-assistants had to write a report. This report gave us insight in the quality of their performance, as it showed that all observations appeared to be rigorously executed.

### Measures

All Facebook profile elements were coded (e.g., profile picture, name, count of friends, interests, textual wall posts, pictures, videos, or notes). This information was coded two times, first when being logged out of the SNS (focus on information accessible for everyone) and secondly after logging into the SNS. For each item, observers had to determine whether this information type could be found on the profile, and if so, to what extent. If possible, coding resulted in a continuous measure (e.g., how many pictures), otherwise in a categorical, but ordinal measure, giving the highest score when most information is given (e.g., profile picture: 4 = recognizable picture of the user, 3 = non-recognizable picture of the user (e.g., group picture, picture taken from far away,..), 2 = a picture, but not of the user (e.g., a cartoon), 1 = no picture).

Moreover, for particular information types, that is interests, pictures, videos and contact information, it was coded if and how much *risky information* was present, for example signs of alcohol abuse, hate messages, etc. (based on literature review, see above). The amount of risky information was coded following a 4-point scale for every single identified indicator of risk (1 = no risk, 4 = a lot of risk). A mean score of all individual risk indicators was calculated for every separate information type to give an indication of the amount of risk in interests (13 indicators, e.g. signs of hate messages), pictures (14 indicators, e.g. signs of nudity), videos (14 indicators, e.g. signs of alcohol use) and contact information (6 indicators, e.g. presence of e-mail address). A general mean score was calculated as well, to give an indication of the total amount of risky information displayed on a profile page. Data are available on http://users.ugent.be/~evderhov/Data_observatiestudie/ after obtaining a username and password from the first author.

### Ethics Statement

The institutional review board, Ethical Committee Psychology and Educational Sciences, approved the research design and waived the need for written informed consent from the participants. Obtaining informed consents would have jeopardized the reliability of the study. Teenagers could have changed their Facebook-profile after being informed about the study before observations took place. However, for ethical reasons it was carefully guarded that the dataset stayed anonymous - by name encryption - and that no personal information was stored. Only the fact that particular information was visible on a profile – and not the information itself - was registered and coded. As such, this research is also aligned within the terms of use of Facebook, and no extra explicit demand for permission was necessary.

## Results

The results are reported following the three research questions. ANOVA was used to study differences in continuously measured variables regarding age, education or gender. To find the effects of age on dichotomous measured variables, a binary logistic regression was used. To find out if education and gender were independent of dichotomous measured dependent variables, *χ* ^2^-tests were carried out. Ordinal regression was used to find the effect of age, education or gender on ordinal dependent variables with more than two categories. Concerning the latter, normality of the distribution was checked and subsequent analysis approaches were adopted [24]. Only *χ* ^2^-statistics (model fit) are reported below. A significance level of *p*<.05 was put forward.

### RQ 1: What kind of information do teenagers post on their Facebook-profile page?

To answer this question, only information on the friends-pages was taken into account, since it is possible that on friends-of-friends-pages not all information was visible because of privacy settings. Table 2 summarizes the information types dominantly present on Facebook profile pages.

**Table 2:** Types of information, percentages and average number on friends' Facebook profiles.

Type of information	Examples	Percentage	M count
Personal information	Correct family name	98%	
	Surname	98%	
	Correct date of birth	80%	
	Correct gender	90%	
Pictures	Self-posted	100%,	298
	In which they are tagged	91%	208
Interests	I like	95%	223
	Music	85%	21
	Movies	65%	4
	Television	76%	8
Videos	Self-posted	35%	
	In which they are tagged	50%	
Wall		47%<10 posts	
Games/applications		49%	
Notes		8%	

Most profiles of friends contained at least personal information such as name, date of birth and gender. The presence of pictures and interests (especially ‘I like’-interests) is proportionally high, while videos, textual wall-posts, games/applications and notes seem to be less popular aspects of Facebook.

It was found that younger children play more games (*χ^2^*(1) = 72.07, *p*<.001) than older ones. Additionally, they post more information about sports (*β* = −.09, *t*(1045) = −2.90, *p* = .004), athletes (*β* = −.08, *t*(1045) = −2.67, *p* = .008) and sports teams (*β* = −.07, *t*(1045) = −2.09, *p* = .037). Older teenagers on the other hand, post more pictures (*β* = .17, *t*(1045) = 5.71, *p*<.001), videos (*χ^2^*(1) = 11.64, *p* = .001) and textual wall posts (*χ^2^*(1) = 6.31, *p* = .012).

Regarding gender, it was found that girls post more pictures, are more tagged in pictures and tag more other persons in pictures than boys (*F*(3,874) = 31.28, *p*<.001). Girls also post more videos (*χ^2^*(1) = 9.99, *p* = .002) and are tagged in more videos (*χ^2^*(1) = 4.44, *p* = .035). Moreover, they post more personal interests (*I likes*) (*F*(1,1044) = 16.35, *p*<.001), have more textual wall posts (*χ^2^*(1) = 13.14, *p*<.001) and include more notes (*χ^2^*(1) = 4.31, *p* = .038). However, boys play more games than girls (*χ^2^*(1) = 9.54, *p* = .002) and more regularly share their mobile number (*χ^2^*(1) = 10.37, *p* = .001) and website, (*χ^2^*(1) = 5.97, *p* = .015). Concerning the education form in which teenagers are enrolled, no significant differences regarding profile content could be found.

### RQ 2: Do teenagers manage privacy settings to secure information?

Privacy settings on Facebook are managed as such that for most information types, one chooses between visibility for friends, friends-of-friends, or everyone. There is also an additional option which makes it possible to differentiate between friends.

To determine information visibility for “everyone”, the profile was analyzed being logged out of Facebook. Since Facebook protects minors by setting their privacy settings to the minimal level of friends-of-friends, information of minors could not be seen. Therefore, we checked profiles of the 18-year olds in our sample (*n* = 182). 63% of their profiles could be accessed without being logged in; e.g., by using Google or the Facebook search engine. In 90% of the cases, profiles revealed their real name and surname, which is actually mandatory on Facebook. Moreover, 70% showed a recognizable picture of themselves on their profile and 73% showed their interests. Other pictures and wall-posts appeared to be better protected and were only accessible in 4% of the cases.

To determine whether teenagers protect their information for friends-of-friends using their privacy settings, the proportion of information visible on friends' pages was compared with the proportion of information accessible on the friends-of-friends' pages. If in general, teens do not change their privacy settings to visibility for friends only, we expect to observe the same proportions of information on the friends' pages as on the friends-of-friends' pages. However, if a significant amount of teenagers changes their privacy settings to visibility for friends only, we expect to observe less information on the friends-of-friends pages. As shown in Table 3, there is no significant difference regarding name, surname or gender. Yet, there is a significant difference regarding pictures, interests, wall posts, videos, e-mail address, relationship status, and date of birth. The percentage of pages of friends-of-friends where this information could be observed was significantly lower compared to the pages of friends, indicating that a significant amount of teenagers set their privacy settings to ‘friends-only’ regarding these aspects. However, if we have a closer look at the actual percentages, we have to conclude that the amount of pages including pictures, interests and textual wall posts accessible for friends-of-friends, is still high (86%, 79% and 48% respectively). This means that although a significant amount of teenagers changes privacy settings to friends-only, another large amount of teens does not protect this information for friends-of-friends.

**Table 3:** Proportion of pages of friends and friends-of-friends that include different types of information.

	Friends	F-of-F	χ^2^(1)	φ
Name	96%	97%	1.94	.04
Surname	90%	90%	.12	.01
Gender	90%	91%	.03	.01
Posted pictures	100%	86%	71.52***	.26
Interests	95%	79%	57.48***	.23
Date of birth	80%	64%	32.10***	.18
Wall	88%	48%	37.13***	.21
Relationship status	58%	38%	42.55***	.20
Posted videos	35%	16%	47.03***	.22
E-mail address	85%	5%	681.85***	.81
Religion	10%	6%	5.48*	.07

χ^2^ tests the significance of the differences in proportions.

*** = *p*<.001,

* = *p*<.05,

Phi's coefficient is given as a measure of effect size.

A comparable pattern could be found regarding age levels and gender. However, relationship status and date of birth are not protected by younger teenagers, aged 13 to 14 (*χ^2^*(1) = 0.94, *p* = .332 and *χ^2^*(1) = 0.01, *p* = .920 respectively), while this information is protected by older teenagers of 15 to 18 years old (*χ^2^*(1) = 48.84, *p*<.001 and *χ^2^*(1) = 47.63, *p*<.001 respectively). No clear effects of education form could be found, implicating that teenagers enrolled in art, vocational, technical and general education use their privacy settings equally.

### RQ 3: Does available information entail particular risks?

The average amount of risky information found on Facebook profiles, as measured by calculating a mean score building on individual risk indicators, was 1.55 (*SD* = 0.36), on a 4–point scale. The mean amount of risk in interests was 1.43(*SD = *0.49), in pictures 1.97 (*SD* = 0.60), in videos 1.31 (*SD* = 0.42) and in contact information 1.34 (*SD* = 0.43). These scores are rather low. However, some risk indicators might be less regular than others, causing the mean score to decrease. A more detailed interpretation of this rather low but non-negligible amount of risky information was therefore derived by calculating percentages of the presence of different risk indicators. The percentages of the most notable risk indicators are summarized in Table 4. The amount of risk represented in pictures and videos is not very high. Moreover, in line with our findings in the previous section, we find that significantly less teenagers show risky pictures to friends-of-friends than to friends (see Table 4 for *χ* ^2^-statistics). However, still 23% are tagged in pictures of themselves partying, 13% in pictures in which they use alcohol and 16% in pictures of themselves in swim-or underwear, while these pictures can be seen by friends-of-friends. Moreover, privacy settings seem to be less used for videos and interests (as can be seen by the *χ* ^2^-statistics and effect sizes in Table 4). Nevertheless, the percentages of risky information displayed in their interests are rather high. A lot of teenagers press the ‘I like’-button in relation to topics about partying, alcohol, bad attitudes toward superiors or school and hate messages. The amount of commercial aspects reflected in their interests also shows the implicit commercial risks they are facing.

**Table 4:** Percentages of risk behavior on Facebook profiles.

	Posted pictures				Tagged pictures				Videos				Interests			
Risky information	F	FOF	χ^2^(1)	φ	F	FOF	χ^2^(1)	φ	F	FOF	χ^2^(1)	φ	F	FOF	χ^2^(1)	φ
Partying	28%	15%	26.54***	.16	55%	23%	114.66***	.33	7%	5%	2.54	.05	47%	35%	15.85***	.12
Alcohol	13%	6%	15.21***	.12	34%	13%	62.22***	.24	3%	2%	1.74	.04	37%	26%	14.85***	.12
Nudity (swim- or underwear)	18%	9%	19.63***	.14	41%	16%	78.83***	.27	2%	1%	2.68	.05				
Bad attitudes directed to school													54%	37%	31.65***	.17
Bad attitude directed to superior													40%	27%	19.82***	.14
Hate messages													37%	31%	4.37*	.07
Commercial messages													41%	35%	5.32*	.07

F = friends, FOF = friends of friends, ***χ*** ^2^ tests the significance of the differences in proportions.

*** = *p*<.001,

* = *p*<.05,

Phi's coefficient is given as a measure of effect size.

The analysis of the nature and amount of private contact information shows that this information is rather scarce. While some information is available for friends – e-mail (85%), instant messenger (23%) - this information is mostly protected from friends-of-friends (e-mail (5%) and instant messenger (1%)). Only the city where they live is not well protected, and can also be found on 43% of the friends-of-friends pages.

An ANCOVA was conducted with gender and education as fixed factors, age as a covariate and the amount of risk as a dependent variable. A significant relationship with age (*F*(1,934) = 72.81, *p*<.001), and a significant gender difference (*F*(1,934) = 7.33, *p* = .007) were found, but there were no differences concerning education form (*F*(3,934) = 1.71, *p* = .163). Older teenagers and girls post more risky information on their profile, but there was no significant interaction between age and gender (*F*(1,933) = 0.24, *p* = .630). Additional ANCOVA's based on the amount of different types of risky information, with age as a covariate and gender as a predictor, show us that older teenagers post more risky pictures, videos, interests and contact information. Girls post more risky pictures, videos and interests than boys, but no significant gender difference is observed in the amount of contact information (Table 5). Moreover, no significant interaction effects could be found. Effect sizes show that all effects found are small to moderate [25].

**Table 5:** Age and gender differences in the amount of risks as related to types of information on their Facebook profile page.

Risks in	Age			Gender			
	*B*	*F(1,1033)*	*Cohen's f*	*Boys M(SD)*	*Girls M(SD)*	*F(1,1033)*	*Cohen's f*
Pictures	1.33	121.66***	.34	21.14(6.57)	22.10(6.96)	9.00**	.10
Video	.99	42.53***	.20	6.82(7.94)	8.22(8.54)	9.80**	.10
Interests	.33	6.09*	.10	17.89 (6.82)	18.70 (7.35)	3.88*	.00
Contact information	.04	6.32*	.10	6.72(.94)	6.66(.78)	1.26	.00

* = *p*<.05,

** = *p*<.01,

*** = *p*<.001.

Cohen's f gives an indication of the effect size [25].

## Discussion

This study extends the results found in previous exploratory research by observing both the public and non-public (i.e., only visible for friends or friends-of-friends) Facebook-profiles of teenagers, a target group which was unrepresented in previous observational research. The objective was to map (1) the nature of information that teenagers post online, (2) their use of privacy settings and (3) the amount of risk that is related to SNS usage by teenagers, by building on the strengths of an observational research design.

As an answer to the first research question, we observed that teenagers post a variety of information types on their SNS-profile, that is mostly pictures, interests and some basic personal information. This can be interpreted in the context of constructing an online identity [26], [27]. This seems to be especially the case for older girls, who seem to post more pictures, interests, wall-posts, etc. Pictures and interests indeed help in building and revealing one's identity [22]. Although this process has always existed, SNSs give the personal and social identity construction a new dimension. The profile pages used to build an identity are often available for more people than just the peers they were built for, thereby complicating the process of privacy protection.

However, privacy can be protected by managing privacy settings in a conscious way. Yet, as an answer to our second research question we found that although a significant amount of teenagers change privacy settings to ‘friends-only’, another large amount of teenagers still reveals a lot of information to friends-of-friends. If we take into account the average number of friends (*M* = 384), friends-of-friends might imply *a lot of strangers*. Still, another way to protect privacy is by selecting the content of a SNS profile page carefully. As an answer to our third research question we observed that teenagers did not post a large amount of contact information on their profile page. This might be the result of the ongoing safety messages that society, peers, parents and teachers give to teenagers: do not make your address or phone number available online! It has been found that - in the European context - parents restrict their children in giving personal information to others, such as contact information (but not pictures, videos,..), and that girls between 13 and 16 years old are more restricted than boys of this age [2]. This can explain why - unlike other risky information - no gender differences were found in the amount of contact information teenagers make available online. However, particular information seems to “slip” teenagers' attention. For example, it was found that the place (town, village) where teenagers lived was visible for friends-of-friends in almost half of the cases. This information can, combined with the name and surname, be sufficient to track detailed contact information. Moreover, we observed that a lot of - potentially risky - information was present on profile pages, such as items referring to alcohol abuse, partying, or nudity. In line with [4], it was also found that older teenagers post more information, and more risky information on their profile page. The last suggests that teenagers care about posting information, but forget to erase information. Moreover, the fact that the management of privacy settings remains restricted - a replication of previous findings [2]- might indicate that teenagers' awareness of privacy risks has not increased over age, and/or that they lack adequate technical skills to manage profile pages in a safer way.

### Observational research versus Survey based research

To answer the three research questions about SNS use of teenagers and their management of privacy settings, an observational research design was used to overcome possible disadvantages of research methods based on self-report. It is therefore interesting to study to what extent the present results differ from previous survey based research. Building on a quantitative self-report study, it was found that 46% showed their name, 86% their surname, 65% posted pictures and 17% had ‘I likes’ on their SNS profile [5]. In the present observational study, higher proportions were found (96%, 98%, 100% and 95% respectively). Moreover, compared to the results previously found in – a survey based -research [18], our results did not confirm the finding that boys share more self-promoting and risky pictures and girls post more romantic or cute pictures. In the present study, we found the opposite: girls tend to post more risky pictures. These divergent findings can possibly be explained by socially desirable answers on surveys by girls, who might not want to admit that they have risky pictures on their profile. It has been found in previous research that girls might be more susceptible to social desirability (e.g., [28]). Moreover, we did not find any differences between pupils enrolled in different education forms, while differences in sharing contact information have been found in survey based research [7]. Again, social desirable answers in the survey research can explain these contradicting findings. The desirability of sharing contact information might be context-related, causing divergent social desirability bias on surveys between pupils enrolled in different education forms. Further research is necessary to entangle the exact reasons for the observed differences in research findings, but they already exemplify the potential disadvantages of self-report based measures.

Focusing on the management of privacy settings, the newly acquired information is more detailed as compared to what can be obtained via surveys. For every information item on the profile page, it could be determined if this was accessible for everyone, friends-of-friends or friends only. This analysis approach is even more detailed as compared to previous observational studies (e.g., [11], [19]), which only focused on public profiles. Our results show for example that a large set of information is still visible on friends-of-friends pages. Our more detailed observational approach might also explain why we did not find gender differences in privacy settings, contradicting previous research [2].

Possibly also because of our focus on non-public profile pages (including pages that could only be seen by friends or friends-of-friends), we could identify higher percentages in risky information as compared to previous research [15], like signs of alcohol abuse. We found that 34% of the friends' profiles had pictures in which they were tagged using alcohol, while also 13% of the friends-of-friends' profiles contained pictures in which they were tagged using alcohol. This is in sharp contrast with previous studies [15] that only found signs of alcohol use in 2% of their observed –public- profile pages. This indicates that our observational methodology might result in more detailed and possibly more discomforting information.

### Limitations

Yet, the results of the present study also have to be considered in the light of some limitations. First of all, the comparison between results of survey studies and this observational study should be interpreted cautiously, since no direct statistical comparisons could be made. Ideally, a follow-up study should compare the results of observation and the results of surveys from the same teenagers. In the current study, this was not possible since the owners of the observed Facebook profiles were completely anonymous, for ethical reasons.

Second, in this study we only observed Facebook profile pages. Although this is currently the most used SNS, different results might be found when focusing on different SNSs. This implies that change in the design of a SNS might cause changes in related behavior we are currently not aware of. The rapidly changing context of SNSs and the corresponding adjustments of the SNS architecture also entail changes in the nature of the risks that teenagers face [29]. Although we tried to be as exhaustive as possible with regard to the coding of risky behavior, examples of risky behaviors that were not included in the current research, but might increase in importance in the future, are the disclosure of medical information (that might be sold to insurance companies) and the use of the Facebook function to reveal one's location (that might invite burglars). Generally, it is hard to predict what the role of SNSs will be in the lives of teenagers in five to ten years, or what SNSs would look like in the future, if they still exist. Therefore, the results of the current research are temporary and will need follow-up studies in the future.

Third, we only observed profile pages of Flemish teenagers. Though our results help to map user profiles of Flemish teenagers, replication studies are needed to validate our findings in broader cultural contexts. Indeed, previous research shows that there might be important cultural differences in people's behavior on Facebook [30]. Especially with regard to disclosure, culture and religious background might have an important impact, not only on behavior but also on the amount of risk associated with the behavior. For example, drinking alcohol and showing nudity in pictures can have a different moral impact in Western countries compared to Arabic regions (e.g., [31]). Therefore, similar studies in countries with a different cultural background are invaluable.

Fourth, a limitation of this observational research is that it does not lead to explanations for the observed facts. For example, we found that older teenagers have more (risky) information on their profile, but there is no way to know why this result was observed. Since time registered on the SNS was not taken into account, this result could mean both that older teenagers are posting more information (e.g., because 18 year olds are of legal age), but also that it was an accumulation of information gathered over time. Future research should focus more deeply on the nature of the relationships that were established in this research.

Finally, although we tried to optimize the randomization of profile page selection, bias could have entered in the selection procedure. However, since we used a stratified random sampling procedure, controlling for age, gender and education form, we remain confident that our sample is representative for Flemish teenagers. Moreover, by involving 179 independent research-assistants we mirror closely a randomized sampling procedure. This way, we could go beyond the limitations of a focus on public profiles only, resulting in an innovative contribution to the literature by presenting information and conclusions about minors, an important and vulnerable group of users of SNSs thus far hardly studied in the literature.

### Implications

Since we found that teenagers still post a lot of personal and risky information on their profile page and they hardly manage their privacy settings, we can conclude that awareness-raising interventions and/or regulatory policies remain necessary. Since in our study no differences were found regarding the education form teenagers were enrolled in, generic interventions should be set up involving teenagers enrolled in all types of education forms. However, the focus of the interventions should be different for different age-groups: 13 to 14 year olds seem to be more vulnerable to commercial risks and privacy risks resulting from third companies (by playing games), while 15–16 year olds are more concerned about building their personal/social identity, and should be alerted to the risks related to the content they post online.

While researchers, parents and policy makers emphasize the role of school education about safety on SNSs [15], [32], [33], research about the impact of interventions shows mixed results. A survey research showed that the attention for risks on SNSs in schools is small, but that it might have an indirect impact on teenagers' behavior by raising privacy care [34]. Moreover, it was found that even a brief e-mail intervention can already redirect online behavior [35]. Still, recent intervention research in secondary education shows that changes in the SNS behavior of teenagers is difficult to achieve with a short-term school intervention [36]. Therefore further research about successful educational approaches within schools remains necessary.

## Supporting Information

Appendix S1 **Supporting information about the research assistants involved in this research.**
(DOCX)
