# Supplementary material for: How Safe Do Teenagers Behave on Facebook? An Observational Study
Source: PLoS One. 2014 Aug 27;9(8):e104036. doi: 10.1371/journal.pone.0104036 (PMC4146465; doi:10.1371/journal.pone.0104036)
Supplement: Appendix S1 — Supporting information about the research assistants involved in this research. (DOCX) [file pone.0104036.s001.docx]

# Supplementary file: Research assistants

As stated in the manuscript, pages of friends and friends-of-friends can only be seen by friends and friends-of-friends, and not by the main researchers. Our sampling method however overcame this problem by involving 179 research-assistants as observers in this study.

These research assistants were bachelor students in educational sciences at Ghent University. Most of them are Flemish and aged about 18-19 years old. The training consisted out of a three hour course, in which the different steps of the analysis procedure were explained extensively. The codebook was given and explained during this course. Moreover, the main researcher’s Facebook profile was coded during this training as an example.

After the training, a website with step-by-step instructions was continuously available for the students. Moreover, the codebook that they had to use, contained step by step instructions about the coding (e.g., “click on ‘information’, do you see the following things on the profile?”). The example-profile of the main researcher was also included in the codebook by means of screenshots. Red circles showed where they had to look to find certain information, e.g.:

“Is the current employer shown? Yes = 1, No =0”

Students were randomly assigned to groups of four research-assistants. Every group carried out the observational analysis of 24 Facebook profile pages of Flemish teenagers. Before they started the observation, they received the ethical research regulations of Ghent University, and had to sign a form that they declared to have read the regulations and to agree with them.

Students also had to write a final research report about their own observations. This report gave us insight about the quality of the observations (however, all research appeared to be rigorously executed). They also had to peer assess each other anonymously, serving as an extra control mechanism.

During the time of the assignment, there were several moments that students could meet the main researcher to ask questions. Moreover, they could always e-mail questions, and frequently asked questions were put on the website.
